# Enhanced Glial Reaction and Altered Neuronal Nitric Oxide Synthase are Implicated in Attention Deficit Hyperactivity Disorder

**DOI:** 10.3389/fcell.2022.901093

**Published:** 2022-06-21

**Authors:** Peng Zhang, Huyue Fang, Chengjian Lou, Shan Ye, Guanghong Shen, Shijia Chen, Nashwa Amin, Benson O. A. Botchway, Marong Fang

**Affiliations:** ^1^ Department of Psychiatry, Affiliated Xiaoshan Hospital, Hangzhou Normal University, Hangzhou, China; ^2^ Department of Neurosurgery, The Fourth Affiliated Hospital, Zhejiang University School of Medicine, Yiwu, China; ^3^ The Second Clinical Medical College, Zhejiang Chinese Medical University, Hangzhou, China; ^4^ The Affiliated People’s Hospital of Hangzhou Medical College, Hangzhou Medical College, Hangzhou, China; ^5^ Institute of Neuroscience, Zhejiang University School of Medicine, Hangzhou, China; ^6^ Department of Zoology, Faculty of Science, Aswan University, Aswan, Egypt

**Keywords:** attention deficit hyperactivity disorder (ADHD), microglia, neuronal nitric oxide synthase, astrogliosis, spontaneously hypertensive rat

## Abstract

Attention deficit hyperactivity disorder (ADHD) has a complex etiology, and its specific causal factors remain to be elucidated. Aberration of nitric oxide synthase (nNOS) and inflammation, together with astrocytic and microglial cells have been continually associated with several neurological disorders, including ADHD. Using spontaneously hypertensive rat (SHR), we investigated the changes in nNOS, inflammatory, microglial and astrocytic markers in the frontal cortex and hippocampus at three different ages: onset of hypertension stage (i.e., 6 weeks after birth of SHR), established hypertension stage (i.e., 12 weeks after birth of SHR) and senescent stage (i.e., 12 months after birth of SHR), and compared with its age-matched normotensive control, Wistar-Kyoto (WKY) rats. A significant upregulation of Iba-1 expression in the senescent stage of SHR was observed. Further, we observed an upregulated nNOS expression in both onset and established stages of SHR, and a downregulated nNOS in the senescent stage. Our study showed an age-related increment of astrogliosis in the cortex and hippocampi of aged SHR. On the basis of our results, alterations in the nNOS and Iba-1 expressions, as well as age-related astrogliosis, may contribute to ADHD pathogenesis.

## 1 Introduction

Attention deficit hyperactivity disorder (ADHD) is a common neurobehavioral disorder accompanied by brain abnormalities, and is characterized by marked attention deficit, short attention span, hyperactivity, or impulsiveness. It is more common in children, with an estimated prevalence of 3%–5% (Polanczyk et al., 2007). However, children are not the only ones who suffer from ADHD. The disorder can progress into adulthood and lead to unstable relationships, poor work or school performance, and low self-esteem (Okie, 2006). The spontaneously hypertensive rat (SHR) is a genetic model and bred from progenitor Wistar Kyoto rat (Aparicio et al., 2019) and is the most commonly used and studied animal model of ADHD (Bucci et al., 2008). SHRs exhibit behaviors similar to those of ADHD patients in a variety of testing paradigms, including increased motor activity, impulsivity, and inattention. Evidences including candidate genes, neurotransmitter dysfunction, neuropathology and pharmacology supports construct validity of SHR (Song et al., 2020). Meanwhile, the dopaminergic and (-)-noradrenaline systems of spontaneously hypertensive rats are hypofunctional, which is one of the important mechanisms in the pathogenesis of ADHD (Bucci et al., 2008).

Nitric oxide is a widely distributed gaseous chemical messenger generated by nitric oxide synthase (Mittleman et al.) from L-arginine (Calabrese et al., 2007), and can be synthesized by three NOS isoforms; neuronal NOS (nNOS), inducible NOS (iNOS), and endothelial NOS (eNOS) (Lind et al., 2017). Nitric oxide acts as a non-conventional neurotransmitter in the CNS, and is involved in the regulation of autonomic functions (Qadri et al., 2003). Furthermore, nitric oxide acts as a vasodilator in the cardiovascular system; thus, alterations in nitric oxide production can affect blood pressure and behavior (Kluknavsky et al., 2016). The neuronal isoforms encoded by NOS1 are the main source of nitric oxide in the CNS. nNOS has been detected in different types of neurons in the cerebellum, hypothalamus, striatum, cerebral cortex, and hippocampus (Domek-Łopacińska and Strosznajder, 2010; Spiers et al., 2019), having a close association with behavioral, memory, cognition, and learning ability. Previous studies have demonstrated that blocking nNOS in the brain may lead augmented systemic mean arterial pressure (MAP) in SHRs in comparison to age-matched WKY rats (Ferrari and Fior-Chadi, 2005). Nitric oxide synthase has been linked to a variety of behavioral disorders, including ADHD. In particular, NOS1 knocked-out mice are more aggressive and impulsive (Volke et al., 1997).

Oxidative stress plays crucial role in the pathophysiology of ADHD. Oxidative stress known as an im-balance between oxidants and antioxidants in the cells. This imbalance due to malfunction of the antioxidant system or by an uncontrolled level of reactive oxygen species (ROS) (Corona, 2020). Brain is the most vul-nerable organ in the body by the oxidative stress which can harm the integrity of neurons. The inflammatory response is a defense mechanism following the oxidative stress, thus, when there is a redox imbalance, the signaling pathways that modulate the immune system are altered, leading to dysregulation of the immune response. ROS could also lead to the activation of astrocytes and microglia. High concentrations of ROS could activate the high secretion of proinflammatory chemokines and cytokines and produce a vicious circle (Alvarez-Arellano et al., 2020).

Neuroinflammation is characterized by changes in microglia, astrocytes, cytokines, chemokines, and related molecular processes within the brain. Neuroinflammation is a key player in psychiatric disorders, such as bipolar disorder, depression, and schizophrenia (Dunn et al., 2019). In the cerebrospinal fluid of ADHD patients, increased pro-inflammatory factor (i.e., TNF-β) and decreased anti-inflammatory cytokine (i.e., IL-4) were detected (Mittleman et al., 1997). The microglia are the central nervous system’s resident immune cells (Réus et al., 2015). Microglia plays an important role in brain damage, neuronal phagocytosis, network connectivity during development, and secretion of inflammatory and anti-inflammatory cytokines (Corona, 2020). Dopamine (specifically, D1R) agonists inhibit microglial activation, concomitant with increased neuronal activity and improved cognitive impairment in ADHD patients (Yokokura et al., 2021). Therefore, targeting the microglia could be an interventional strategy for ADHD.

Astrocytes have unique functional and morphological characteristics. In ADHD, lack of lactic acid produced by the brain’s astrocyte can cause ADHD hyperactivity (Russell et al., 2006; Medin et al., 2019). Moreover, astrocyte can promote the induction and progression of inflammatory states, which are significantly associated with disease status or severity (Corona, 2020). TNF-α, ROS and other inflammatory stimuli induce expression of NF-κB in the astrocytic nucleus and aggravate neuroinflammation (Linnerbauer et al., 2020). Also, the MAPK/NF-κB signaling minimizes the activation of astrocyte and microglia, and lowers the expression of IL-1, TNF-α and other inflammatory factors in ADHD (Song et al., 2020).

The SHR is the most commonly used and studied animal model of ADHD. This is because SHRs exhibit behaviors similar to those of ADHD patients, including increased motor activity, impulsivity, and inattentiveness (Bucci et al., 2008). More importantly, the hypofunctionality of dopaminergic and noradrenaline systems, which are key mechanisms in ADHD pathogenesis, are evident in SHRs (Bucci et al., 2008). In this study, SHRs were divided into three groups based on phases: the onset of hypertension (6 weeks), established hypertension (12 weeks), and the senescent stage (12 months). Alterations in nNOS expression, activation of microglia, and astrogliosis in the brain at each stage were investigated and analogized with its age-matched normotensive control, Wistar-Kyoto (WKY) rats, from which the SHRs were originally derived (Ganten et al., 1991).

## 2 Materials and Methods

### 2.1 Animals

Six-week-old, twelve-week-old, and twelve-month-old SHR, along with age-matched Wistar Kyoto rats (Beijing Vital Rivor Laboratory Animal Technology Co. Ltd., Beijing) were used. Rats were housed under a 12 h light/dark cycle with free availability to food and water. Experiments were carried out following the NIH Guide for the Care and Use of Laboratory Animals.

### 2.2 Open Field Test

Sixty rats were used, with ten rats from each age group of SHR and WKY. The animals were placed in a square apparatus (100 cm × 100 cm × 45 cm) with black walls on all sides for 5 min. The apparatus was placed under a camera and adjusted. Using the ANY-maze software, set-up programs were calibrated to the recorded areas. Each rat was placed in the center and allowed to move freely. The movement and total distance of rats were recorded during the test time. The arena was cleaned with 75% ethanol after every trial to avoid affecting the test of the subsequent rats.

### 2.3 Tissue Preparation

Before their death, six rats from each age group of SHR and WKY were anesthetized with pentobarbital sodium (50 mg/kg, i.p.), and perfused intracardially with 300 ml of cool normal saline, followed by 500 ml of 4% paraformaldehyde in 0.1 M phosphate buffer (PBS, pH 7.4). The whole brain was carefully removed and dissected. Brain tissues were post-fixed at 4°C in the same fixative overnight and then transferred to 30% sucrose in PBS until the tissue dropped to the bottom of the container. Brain tissues were cut into five sets of serial coronal sections with a cryostat at −20°C. Each fifth section was collected in each group for free-floating immunohistochemistry and immunofluorescence labeling. 20 μm-thick sections were mounted on gel-coated slides and cover-slipped with Permount (Fisher Scientific, Pittsburgh, PA).

Twelve rats from each age group of SHR and WKY were sacrificed by decapitation, and brains were carefully harvested. The frontal cortex and hippocampus were dissected and immediately frozen in liquid nitrogen for Western blotting and reverse transcriptase-polymerase chain reaction (n = 6 per age group).

### 2.4 Immunohistochemistry and Immunofluorescence Labeling

All manipulations were performed in 24-well culture plates at room temperature. Sections were incubated in 0.01 M PBS with 3% H_2_O_2_ and 10% methanol for 5 min to quench the endogenous peroxidase, followed by blocking in 5% normal goat serum in 0.01 M PBS. Sections were then incubated in primary rabbit polyclonal anti-nNOS (1:500; Santa Cruz Biotechnology, Santa Cruz, CA) overnight at 4°C. After a thorough wash in PBS, sections were incubated with biotinylated donkey biotinylated goat anti-rabbit IgG (1:400; Vector Laboratories, Burlingame, CA) for 1 h at room temperature, followed by an avidin-biotin-peroxidase complex (Vectastain Elite ABC Kit, 1:300, Vector Laboratories, Burlingame, CA) for 1.5 h at room temperature. The reaction product was developed with 0.025% 3,3-diaminobenzidine (DAB) and 0.0033% H_2_O_2_ in 0.2 M Tris-HCl (pH 7.6), and the reaction stopped by rinsing the slides with 0.2 M Tris-HCl. Sections were mounted onto 0.02% poly-L-lysine-coated slides, allowed to dry at room temperature, dehydrated through graded series of alcohol, cleared in xylene, and finally cover-slipped.

For immunofluorescence labeling, sections were permeabilized and blocked with 0.3% Triton X-100 and 10% normal goat serum in 0.01 M PBS, incubated in primary antibodies against nNOS overnight at 4°C, incubated with 1:500 Alexa Fluor 488 Donkey anti-rabbit IgG (Molecular Probes) secondary antibodies for 1 h at 37°C, and finally coverslipped with antifade gel/mount aqueous mounting media (Southern Biotech, AL, United States). All negative control sections were incubated in PBS without primary antibodies. Immunofluorescence labeling neurons were photographed with a confocal imaging system (the Bio-Rad MRC-1024UV system, United Kingdom) connected to a Zeiss Axiophot microscope. The digital images were processed using confocal assistant software (Bio-Rad, Richmond, CA).

### 2.5 Immunofluorescence Staining

After anesthesia, rats were perfused with 1% PBS and 4% PFA. The brain was quickly removed from the skull, soaked in 4% PFA, and fixed at 4°C for 24 h. Then remove paraformaldehyde and add 30% sucrose solution for dehydration until the brain sinks into the bottom of the solution. OCT was used to embed and make continuous frozen sections at 20 nm. Serial consecutive sections were processed for immunofluorescence staining to detect GFAP as a marker of astroglial reaction, Iba-1 as a microglia marker. Collect and pick out the complete brain slices, wash off the embedding agent with PBS solution, 3 times / 5 min. Then, after blocking with blocking solution for 1 h, primary antibodies Iba-1 (Abcam, 1:500), GFAP (Santa Cruz, 1:100) and TNF-α (Abmart, 1:400) were added and incubated overnight at 4°C; Then wash with PBS 3 times for 5 min each time. Add secondary antibody solution and incubate at 37°C away from light for 1 h; Wash 3 times with PBS for 5 min each time and seal with DAPI (VECTASHIELD, United States) containing sealant. Then it was observed with a fluorescence microscope (Olympus BX51, Tokyo, Japan) at an excitation wavelength from 547 to 570 nm (Cy3, red), 494–520 nm (FITC, green), and 360–460 nm (DAPI, blue). Images were taken at 200x magnification.

### 2.6 Reverse Transcriptase-Polymerase Chain Reaction

Total RNA was extracted with an RNA isolation reagent, Trizol TM (Invitrogen, Carlsbad, CA). Quantitation of RNA was performed by measuring the absorbance of each sample at 260 nm. Quality check was done by gel electrophoresis. RNA samples (2 µl) from each age group were used to generate cDNA. Reverse transcription was carried out using Super-Script ™ III Reverse Transcriptase (Invitrogen, Carlsbad, CA). Thermocycling was performed with a gradient thermocycler (Takara, Japan) using GoTaq^®^ Flexi DNA Polymerase (Promega, Madison, WI). β-actin was used as the internal control. The primers used were: nNOS, 5′-acc agc tct tcc ctc tag cc-3′ (forward), 5′-cct ttg ttg gtg gcg tac tt-3′ (reverse), size of amplicon was 304 bp; β-β-actin, 5′-tgt tac caa ctg gga cga ca-3′ (forward), 5′-aag gaa ggc tgg aaa aga gc-3′ (reverse), size of amplicon was 573 bp.

The PCR reaction started at first by denaturing cycle at 94°C for 5 min, followed by an amplification profile of 35 reaction cycles with denaturation at 94°C for 30 s, primer annealing at 55°C for 30 s, and elongation at 72°C for 1 min. PCR was performed simultaneously on SHR and WKY samples, with internal controls running in parallel. All RT-PCR experiments included negative controls, in which template RNA or reverse transcriptase was omitted. Each PCR product (10 μl) was electrophoresed on a 2% agarose gel containing Ethidium bromide. Resultant gel bands were visualized in a UV-transilluminator, with the optical density of the bands determined with a MultiImageTM II light cabinet (DE-500) that was equipped with Fluochem Beta 1.1 software (Alpha Innotech Corp., CA). The relative amount of the targeted gene was expressed as optical density relative to that of the β-actin.

### 2.7 Western Blot Analysis

Total proteins were extracted from brains with 2 mM PMSF in 1 ml ice-cold RIPA buffer added protease inhibitor cocktail EDTA-free. The protein concentrations were determined using the Bradford protein assay. SDS-PAGE was performed on 12% polyacrylamide slab gel, electrophoresed in Tris-glycine buffer under denaturing conditions, and transferred to PVDF membrane at 70 V for 1.5 h at 4°C in a Bio-Rad TransBlot apparatus. Membranes were blocked for 1 h at room temperature with 5% (wt/vol) nonfat milk in Tris-buffered saline 0.05% (vol/vol) Tween 20 (TBS-Tween; 50 mM Tris base, 200 mM NaCl, 0.05% Tween 20). Membranes were then incubated for 12 h at room temperature with primary polyclonal rabbit anti-GFAP (1:500, Thermo Fisher Scientific, Waltham, MA).

Membranes were rinsed thrice (each wash lasting 10 min) with TBS 0.05% Tween 20 (TBST), incubated at room temperature for 1 h with secondary horseradish peroxidase (HRP)-conjugated goat anti-rabbit IgG (1:5,000, Santa Cruz Biotechnology, CA) and then washed again with TBST. Proteins were detected by enhanced chemiluminescence (ECL reagents; Amersham, Piscataway, NJ) exposed to radiographic film (Hyperfilm ECL; Amersham) as described in the technical manual provided by Amersham Pharmacia Biotech, Inc. Densitometry of the immunoreactive bands of interest was performed by acquiring digital images of the radiographic film (Hyperfilm ECL; Amersham) using Bio-Rad Quantity One 1-D software. To normalize protein bands to a gel loading control, membranes were washed in TBST and re-probed with rabbit anti-β-actin (1:5000, Abcam, Cambridge, MA) followed by incubation with peroxidase-conjugated goat anti-rabbit (1:5000, Santa Cruz Biotechnology, CA) and ECL detection. For the negative control, the primary antibody was omitted.

### 2.8 Statistical Analysis

Data from morphometric studies of immunohistochemistry, Western blot analysis, and RT-PCR were presented as mean ± SD. Data from the open field test were presented as mean ± SEM. One-way analysis of variance (ANOVA) with post hoc Tukey tests was used to determine statistical significance. *p* value of <0.05 was considered statistically significant. All statistical tests and graphs were performed or generated using GraphPad Prism Version 8.0 (GraphPad Prism Software, Inc., CA, United States).

## 3 Results

### 3.1 Spontaneously Hypertensive Rat Display Behavior Paradigm of Attention Deficit Hyperactivity Disorder

**FIGURE 1:**
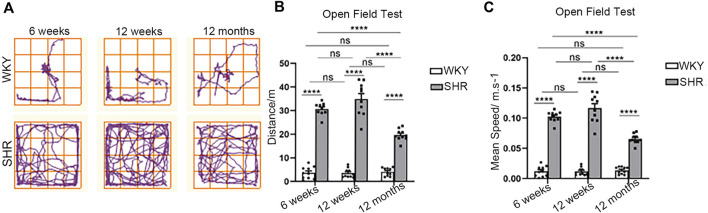
Open field test in WKY and SHR rats. **(A)** The typical trajectory of the WKY and SHR rats in OFT. **(B)** Total distance traveled in the OFT. ****p < 0.0001; ns = not significant. n = 10. **(C)** Mean speed in the OFT. ****p < 0.0001; ns = not significant. n = 10. The values represent mean ± SEM.

The open-field test showed that the locomotor trajectory, total distance and mean speed of SHRs were significantly different from WKY rats. Regarding the activity trajectory (Figure 1A), SHR rats had a wider range of activity and more complex trajectories, whereas WKY rats had a simple and single route with a smaller range and more reciprocal movements. The total distance was significantly higher in SHRs at all ages compared with WKY rats (Figure 1B) in each age group (*p* < 0.0001.) Also, the mean speed of SHRs in each age group was significantly faster (*p* < 0.0001 when compared with WKY rats in the same age group.

The total distance and mean speed of rats in the WKY groups were relatively stable and did not vary with age. In contrast, there was a significant time dependence in the SHR group rats. From weeks 6–12, there was gradual increase of blood pressure (Vilskersts et al., 2014), coinciding with higher distance and faster average speed. Nonetheless, this difference was insignificant (*p* = 0.0913, *p* = 0.0912). At 12 months, the distance and speed were significantly decreased when compared to 6- and 12 week-old rats (*p* < 0.0001, *p* < 0.0001).

### 3.2 mRNA Expression of Neuronal Nitric Oxide Synthase is Increased in the Frontal Cortex and Hippocampus of Spontaneously Hypertensive Rat

**FIGURE 2:**
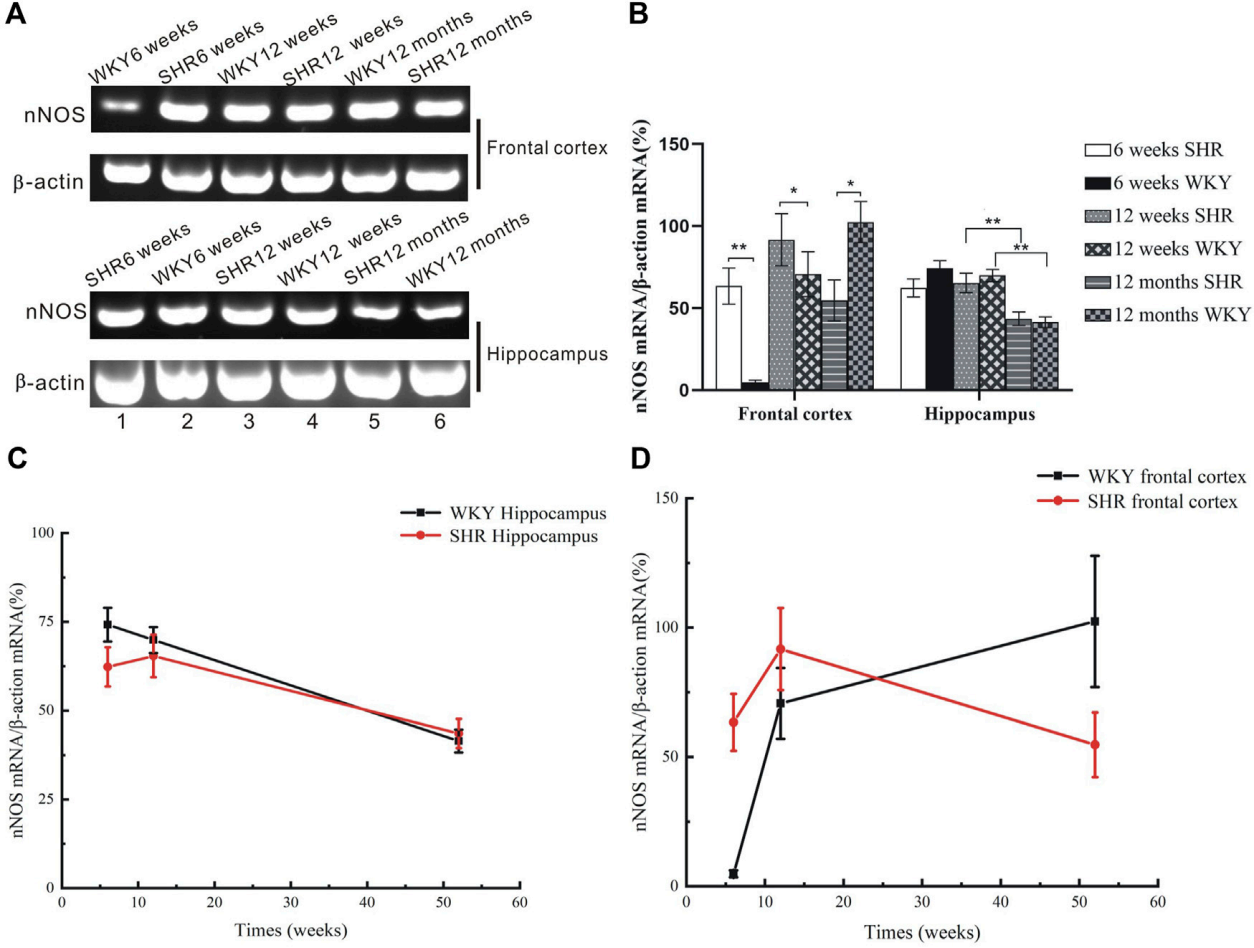
mRNA expression of nNOS from the frontal cortex and hippocampus of SHR and WKY. **(A)** Agarose gel electrophoresis of PCR-amplified products nNOS mRNAs in the hippocampus of SHR and WKY. The location of bands for each of the products corresponded to the expected simplified cDNA fragment size based on the choice of oligonucleotide primers. **(B)** The expression of nNOS was upregulated at 6- and 12 weeks postnatal stage but decreased at 12 months in the SHR, while the nNOS expression increased at the senescence stage in WKY. *p <0.05 verse age-matched WKY control, **p <0.01 versus age-matched WKY control. **(C)** The change of nNOS expression in the frontal cortex in aged process of SHR and WKY. **(D)** The change of nNOS expression in the hippocampus in aged process of SHR and WKY.

**FIGURE 3:**
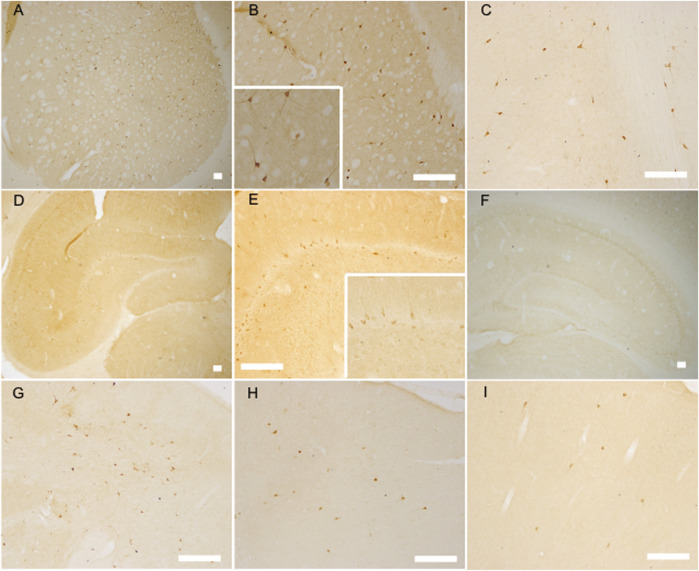
The nNOS positive labeled neurons increased at the established hypertension stage (12 weeks) but decreased at the senescence stage (12 months) of SHR as analyzed by nNOS immunoreaction. nNOS positive labeled neurons in the striatum of 12 week-old SHR **(A,B)** (with an amplificatory image inserted into B) and WKY **(C)**, the hippocampus of 12 week-old SHR **(D,E)** (with an amplificatory image inserted into E) and WKY **(F)**, the frontal cortex of 12-week-old SHR **(G)** and WKY **(H)**, the frontal cortex of 12 month-old SHR **(I)**, bar = 100 μm.

At weeks 6 and 12 post-natal stage we found higher levels of nNOS mRNA levels in frontal cortex in SHRs than WKY rats (Figures 2, 3, *p* < 0.05). The expression of nNOS in 12 weeks SHR was higher than that in 6 weeks SHR. WKY rats also showed the same trend. However, the mRNA level of nNOS was considerably increased in the aforementioned brain regions of aged WKY (12 months old) but declined substantially in the age-matched SHRs (Figures 2, 3G,I *p* < 0.01).

### 3.3 GFAP Expression Increases Gradually With Age

**FIGURE 4:**
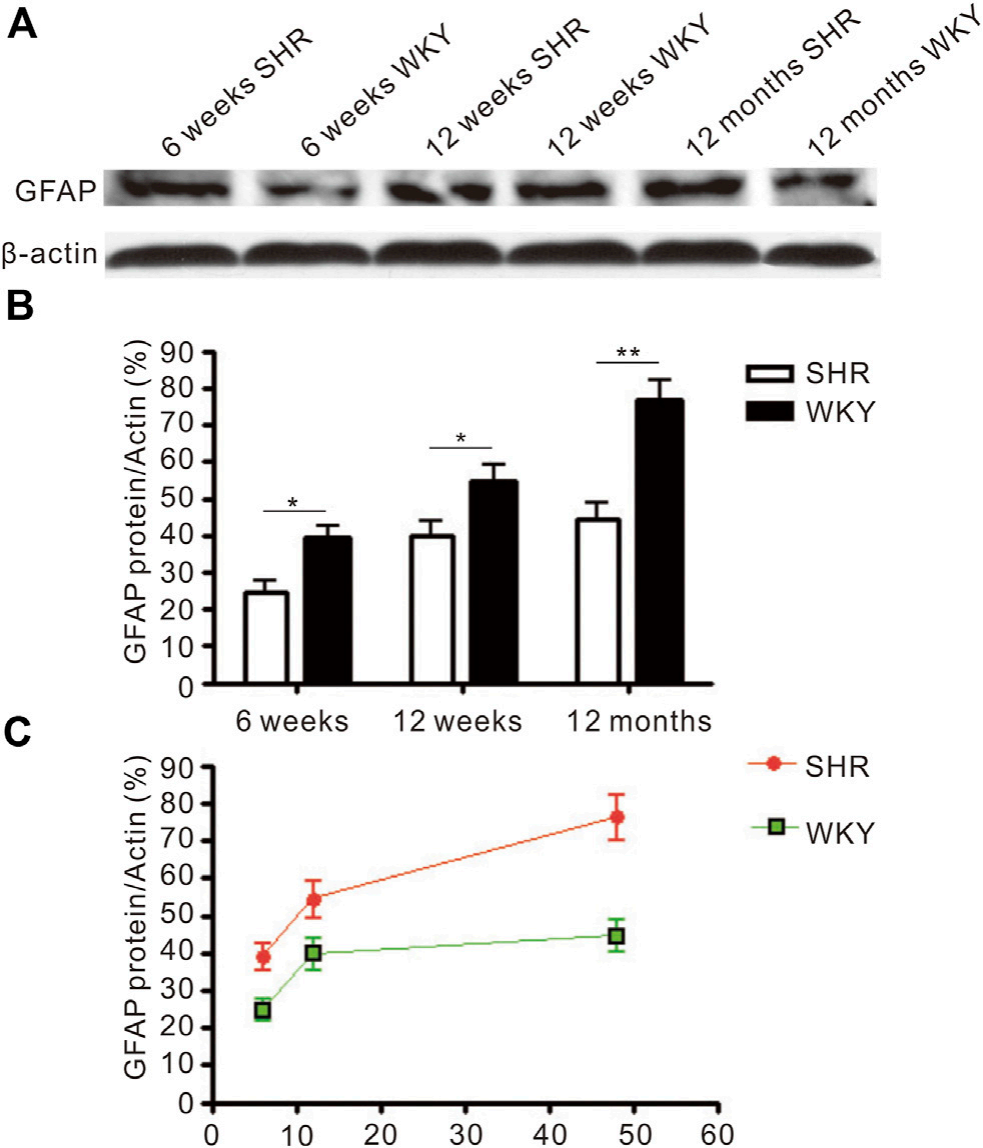
The level of GFAP protein expressed in the frontal cortex. **(A)** The Western blot result of GFAP in the frontal cortex of SHR and WKY. **(B)** The increased expression of GFAP in the frontal cortex was more severe in the SHR than in WKY. *p < 0.05 versus age-matched WKY control, **p < 0.01 versus age-matched WKY control. **(C)** The change of GFAP expression in the frontal cortex in aged process of SHR and WKY. The values represent mean ± SD.

Immunoblotting analysis showed the protein level of GFAP was increased with age in the frontal cortex and hippocampus of SHR and WKY rats (Figure 4). Higher GFAP protein level was detected in the brain of SHR when compared with WKY rats at 6 and 12 weeks after birth (*p* < 0.05, Figures 4B,C). Additionally, there was a significant difference in GFAP level at 12 months (*p* < 0.01, Figure 4).

### 3.4 The Expression of GFAP Increases in the Frontal Cortex and Hippocampus of Spontaneously Hypertensive Rat

**FIGURE 5:**
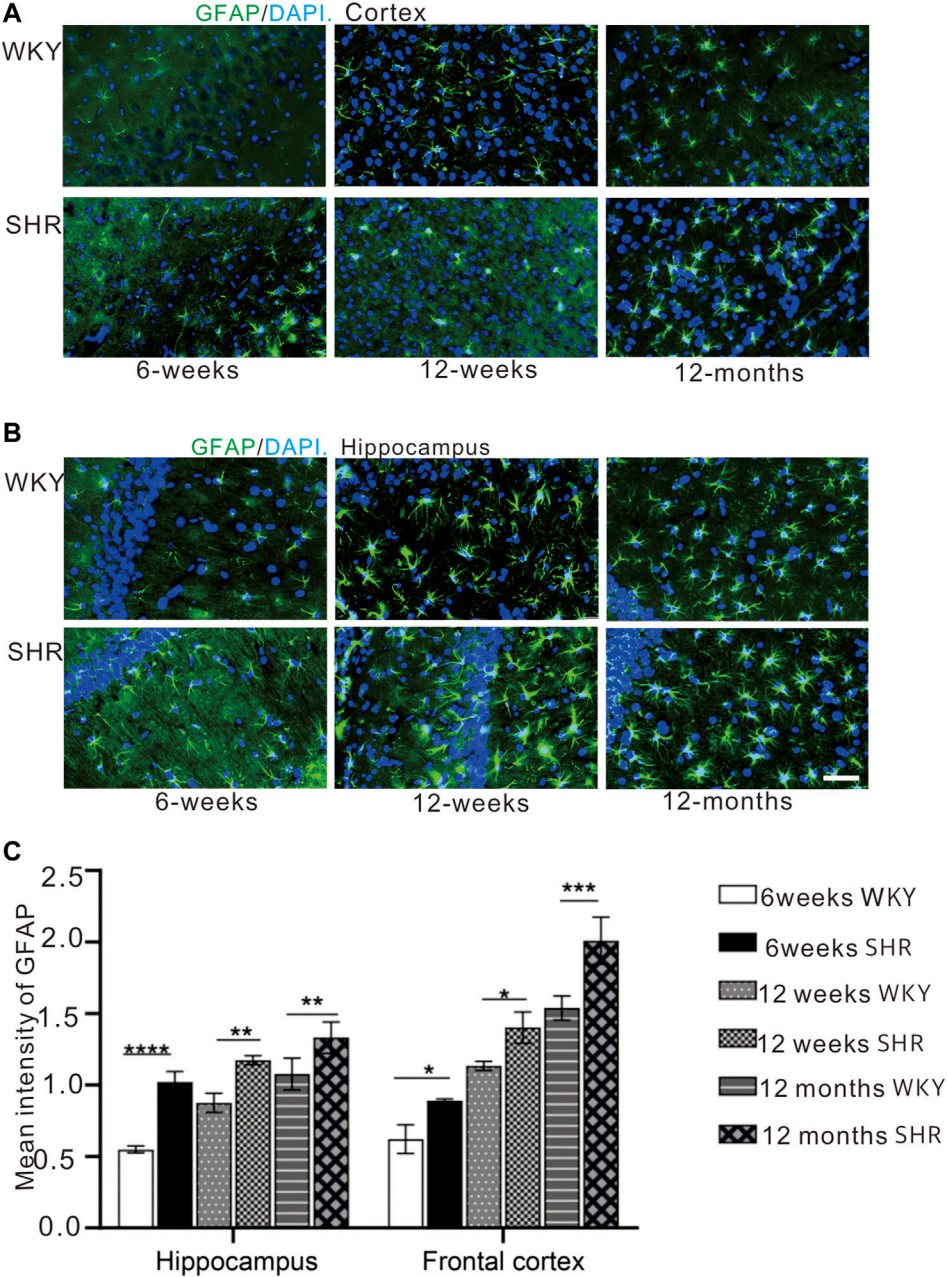
GFAP immunofluorescence showed that the expression of GFAP in the cortex and hippocampus of SHR rats was increased compared with that of WKY rats. **(A)** the expression of GFAP in prefrontal cortex of WKY and SHR, **(B)** the expression of GFAP in hippocampal CA1 of WKY and SHR, bar = 20 μm *p < 0.05 versus age-matched WKY control. **p < 0.01, age-matched WKY control. ***p < 0.001 age-matched WKY control. The values represent mean ± SD. **(C)** The mean intensity of GFAP was upregulated in the SHR at each age-matched group in the hippocampus and frontal cortex.

Immunofluorescence staining (Figures 5A,B) was used to evaluate the expression of astrocyte and the immunofluorescence intensity of GFAP positive cells (Figure 5C). The GFAP expression was increased in the hippocampus and prefrontal cortex of SHR and WKY rats. Also, the GFAP expression was significantly higher in in the CA1 region of SHR hippocampus compared with the WKY group at 6 weeks (*p* < 0.001, Figure 5C). Further, the GFAP expression was increased in other periods when compared with the WKY group (*p* < 0.01, Figure 5C). In addition, the GFAP expression was significantly increased in the SHR group at 12 months (aging) compared with the WKY group in the prefrontal cortex (*p* < 0.05, Figure 5C).

### 3.5 The Expression of Iba-1 Increases Gradually in the Frontal Cortex and Hippocampus of Spontaneously Hypertensive Rat

**FIGURE 6:**
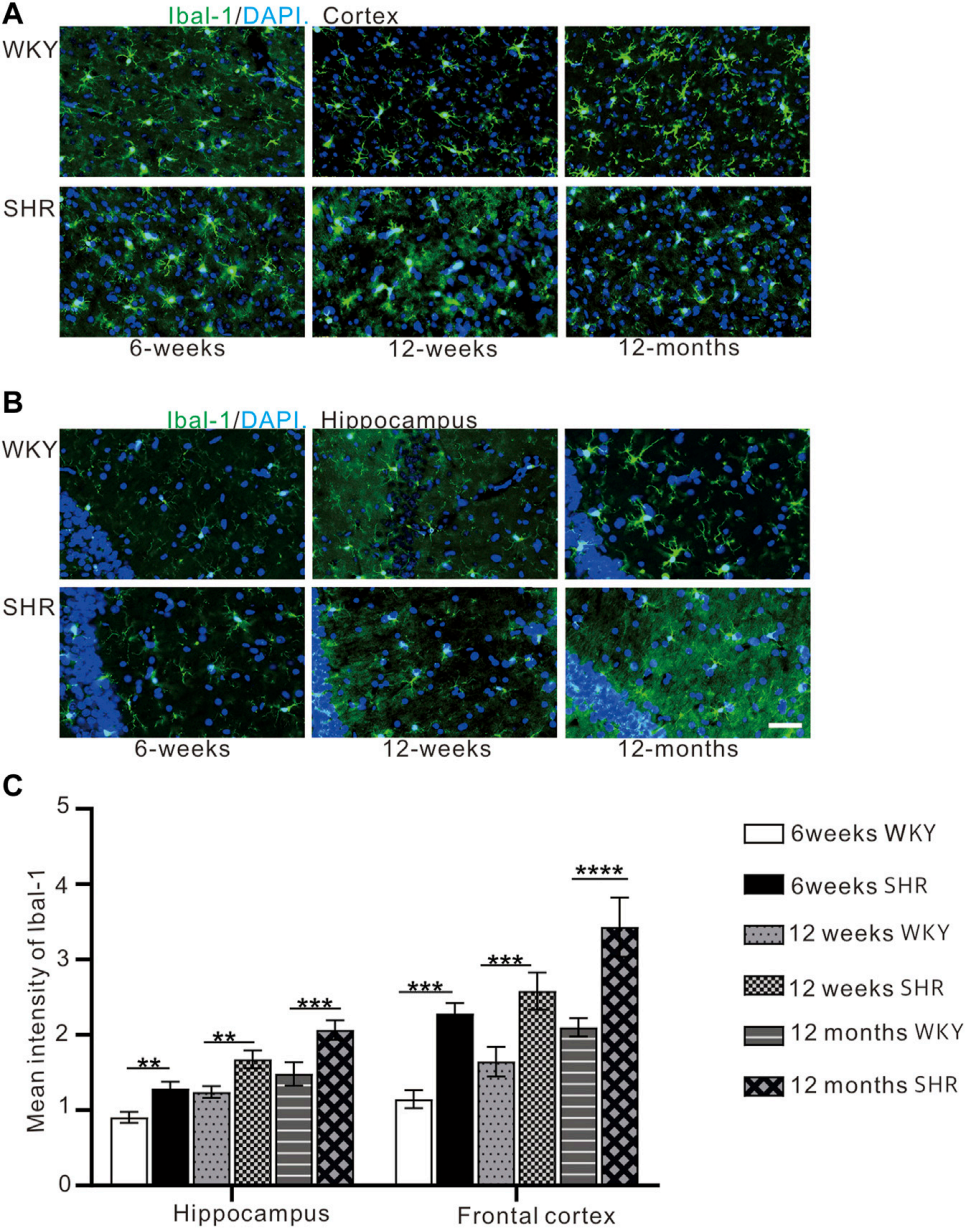
**(A)** The expression of iba-1 in the prefrontal cortex of WKY and SHR. **(B)** the expression of iba-1 in the hippocampal CA1 region of WKY and SHR. **(C)** Iba-1 immunofluorescence showed that the expression of iba-1 protein in the cortex and hippocampus of SHR was increased compared with that of WKY Group, bar = 100 μm. **p < 0.01 versus age-matched WKY control. ***p < 0.001, vs. age-matched WKY control. The values represent mean ± SD.

Immunofluorescence staining (Figures 6A,B) was used to detect microglial activation in the hippocampal CA1 region and the prefrontal cortex. Iba-1 expression was significantly higher in the hippocampal CA1 region and prefrontal cortex region of SHR compared with WKY rats at 12 months (*p* < 0.001, Figure 6C). In comparison with WKY group at 6 and 12 weeks, Iba-1 expression was increased in SHR at the same time (*p* < 0.01, Figure 6C).

### 3.6 The Expression of TNF-α Increases Gradually in the Frontal Cortex and Hippocampus of Spontaneously Hypertensive Rat

**FIGURE 7:**
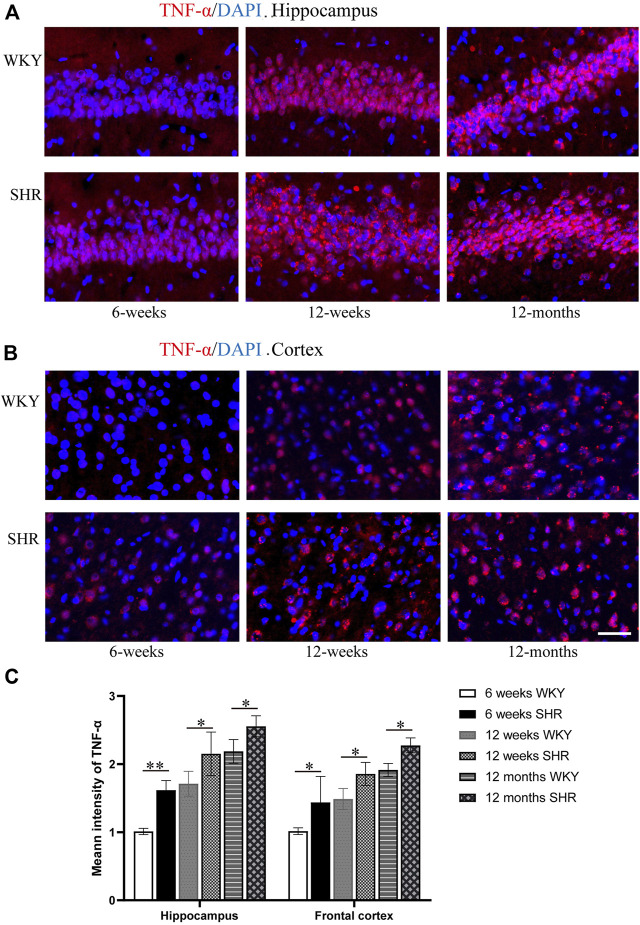
TNF-α immunofluorescence showed that the expression of TNF-α protein in the hippocampus and frontal cortex of SHR was increased compared with that of WKY group, bar = 100 μm. **(A)** The expression of TNF-α in the hippocampus CA1 region of WKY and SHR. **(B)** the expression of TNF-α in the frontal cortex of WKY and SHR. **(C)** The mean intensity of TNF-α was upregulated in the SHR at each age-matched group in the hippocampus and frontal cortex. *p < 0.05 versus age-matched WKY control. **p < 0.01, versus age-matched WKY control. The values represent mean ± SD.

Immunofluorescence staining (Figures 7A,B) was used to evaluate the expression of TNF-α and the immunofluorescence intensity of TNF-α positive cells (Figure 7C). The TNF-α expression was increased in the hippocampus and prefrontal cortex of SHR rats when compared with the age-matched WKY rats. Moreover, TNF-α expression in the CA1 region of the hippocampus was significantly higher than the WKY group at 6 weeks (*p* < 0.01). The TNF-α expression also increased in other periods compared with WKY group (*p* < 0.05). In addition, the TNF-α expression was significantly upregulated in the SHR group compared with the WKY group in the prefrontal cortex of each periods (*p* < 0.05, Figure 7C).

## 4 Discussion

In the present study, SHRs (an animal model of ADHD) were obtained by selective breeding of WKY rats (control animals) with the highest blood pressure. These animal strains are normotensive at birth, and gradually develop stable hypertension during the first weeks of life (Rubattu et al., 2018). The blood pressure of SHR rises around 5–6 weeks of age, and steadily increases to reach systolic blood pressure of 180–200 mmHg. SHR has several features of hypertensive organ damage, including cardiac hypertrophy, cardiac failure, and renal dysfunction in the latter stages of their life. As such, this strain has been employed for evaluating genetic factors in hypertension (Doris, 2017). Besides, SHRs have been used as an animal model for studying vascular brain damage and related pathogenic development of learning and memory deficits (Hussein et al., 2005; Li et al., 2009; Tayebati et al., 2012; Leffa et al., 2019).

The open field test (OFT) is a common experimental method for evaluating the ability of animal self-motion, and is used in multidisciplinary experimental studies. In behavioral studies of ADHD, the distance and speed of movement of rats in the open field test are often used as an important basis for evaluating the two core symptoms of ADHD: hyperactivity and impulsivity. Generally, locomotion is used to assess hyperactive behavior, and the speed of movement is used to evaluate the state of agitated impulsivity in animals.

In this study, SHRs showed marked differences in locomotion when compared to the WKY rats. After 6 weeks, SHRs demonstrated considerable long motor distance and fast motor speed, which are typical of hyperactive and impulsive behaviors, and are behavioral features of ADHD.

Centrally produced nitric oxide affects autonomic responses and participates in the regulation of sympathetic tone to the periphery (Han et al., 2010). Our study found upregulated nNOS expression in the frontal cortex and hippocampus at the onset and established stages of hypertension. This was consistent with a previous study that established gene expression of nNOS was increased in central autonomic centers in animals with increased sympathetic activity. The above statement supports the hypothesis that nitric oxide plays an important role in the maintenance of homeostatic balance through the modulation of sympathetic activity (Hojná et al., 2007). Thus, during over-activity of the sympathetic system in hypertensive condition, the sustained increase in blood pressure and related functional alteration in the autonomic system might act as a stimulus for exacerbate nNOS activity, and nitric oxide production in spontaneously hypertensive rats might be enhanced in CNS to compensate for the elevated blood pressure (Hojná et al., 2007; Sun et al., 2019). However, the excess amount of nitric oxide, if not balanced by antioxidants, will react with superoxide (O_2_^−^) and other reactive oxygen species to produce peroxynitrite, and trigger oxidative stress. Noteworthy is that oxidative stress is involved in neurodegenerative diseases owing to its free-radical properties (Colas et al., 2006; Török, 2008). Besides, it has been correlated with the pathophysiology of ADHD (Alvarez-Arellano et al., 2020).

In the aged animals (i.e., 12 month-old), the brain nNOS expression of WKY rats was significantly higher. This was in line with a previous study that showed that aged animals exhibit increased in cortical NOS activity regardless of their genetic background (Law et al., 2003). Nitric oxide has been implicated in neurodegenerative diseases due to its oxidative properties. The upregulation of nNOS expression in aged WKY might be due to the abatement of free radical scavengers and diminished anti-oxidative capacity in old animals. Thus, elevated nitric oxide release would increase oxidative stress, and coincide with cognitive impairments owing to the accumulation of oxidative damages. Concerning aged SHR, since considerable upregulated nNOS in the early stages of the aging process affects the microenvironment in each sub-regions of the CNS, the secondary change in oxidative stress, excitotoxicity, energy depletion, neuroinflammation together with apoptosis could lead to significant loss of nNOS expressing neurons. The diminution of nNOS positive neuron number in the brain of aged SHR, as evidenced from our experiments, was consistent with this hypothesis, which might, at least in part, explain the decline tendency of nNOS expression in the older SHR.

Oxidative stress in ADHD causes an inflammatory response that affects ADHD attention deficit and hyperactivity disorder (Corona, 2020). Under different pathological conditions, abnormal induction of nNOS leads to excessive production of NO, and results in oxidative stress. ROS can cause microglia activation (Kim et al., 2019). Iba-1 expression was significantly higher in 12 months SHR than the WKY rats. This suggests significant microglia activation in aged hypertensive rats, which may be attributed to the accumulation of oxidative damage. The nNOS expression in the 12 month-old SHRs confirmed this theory. Activated microglia release superoxide anion free radicals, nitric oxide, TNF-α, IL-1 and other inflammatory factors that exacerbate inflammatory damage (Réus et al., 2015). TNF-α expression was higher in 12 months SHR than WKY rats. Compared with the WKY rats, SHR rats at 6 and 12 weeks showed the same trend. High levels of proinflammatory cytokine can affect synaptic plasticity and neurogenesis (Anand et al., 2017; Leffa et al., 2018). Therefore, cytokines can affect cognitive function. This was consistent with dementia behavior in aged hypertensive rats. In addition, the TNF-α is up-regulated in tryptophan metabolism, which is associated with the severity of ADHD symptoms (Aarsland et al., 2015; Anand et al., 2017).

Astrogliosis is an increase in the size of specific cytoskeletal protein GFAP-immunoreactive astrocytes. It is common in several brain damage (Tomassoni et al., 2004). A higher GFAP expression has been reported in several neurodegenerative disorders and autogenic senescence-accelerated mouse (SAM) model (Tomassoni et al., 2004; Wu et al., 2005; Tayebati et al., 2012). The increased astrocyte-neuron contacts could restrict or reduce synaptic plasticity by decreasing the potential interneuronal contacts (Finch, 2003). Our results showed GFAP-positive astrocytes increased with age in both the frontal cortex and hippocampus of SHR and WKY, suggesting it is an age-related pathological change of CNS. We also speculated that an increase in astrocyte volume might lead to a corresponding decrement in neuron volume. Our results from the present study showed GFAP-positive astrocytes to increase with age in the frontal cortex and hippocampus of SHR and WKY rats, suggesting it is an age-related pathological change of the CNS. Also, we speculated that an increase in astrocyte volume might lead to a corresponding decrement in neuronal volume. Our results showed the brains of the SHR model had more severe astrogliosis conditions than the WKY rats, especially at the senescence stage of 12 months, which was incongruity with marked loss of nNOS neurons at this stage. This implies that elevated nitric oxide production in the early stage and early onset of astrogliosis occurring in the brain of animals with established hypertension could hinder the normal survival conditions of functional neurons, and lead to abnormalities, such as impaired learning and memory.

It is worth noting that there are some limitations regarding the use of SHR as a disease model for ADHD. With reference to our present study, the model was selectively bred, and so is difficult to separate the effects of hypertension from hyperactivity (Majdak et al., 2016). In addition, ADHD has been associated with dysfunction in monoamine neurotransmitters pathways, such as 5-hydroxytryptamine (5-HT), dopamine (DA), and norepinephrine (NE) (Klein et al., 2017). Particularly, 1 month after the birth of SHRs, brain expressions of tyrosine hydroxylase and DAT genes are decreased, dopamine and norepinephrine levels are abnormal, and decrease in function exhibits signs of ADHD (Russell, 2007). In their adulthood, expression of tyrosine hydroxylase and DAT genes and monoamine neurotransmitter levels increase, causing a decrease in ADHD symptoms, such as curtailed hyperactive and impulsive behaviors. The specific reasons for the decrease in the distance and speed of senile SHR need to be further studied.

## 5 Conclusion

The imbalanced expression of nNOS, abnormal production of nitric oxide, microglia activation, and age-related astrogliosis that are present in specific brain sub-regions related to learning ability and memory formation may contribute to pathological changes in the brain of hypertensive animal model, ultimately leading to memory and cognitive impairments in ADHD. Investigations targeting the neuronal toxic effects of excess nitric oxide, alleviating age-related astrogliosis processes, and preventing the loss of cholinergic neurons could potentially be an effective interventional medium for ADHD patients.

## Data Availability

The original contributions presented in the study are included in the article/Supplementary Material, further inquiries can be directed to the corresponding author.

## References

[B1] Aarsland T. I. M. Landaas E. T. Hegvik T.-A. Ulvik A. Halmøy A. Ueland P. M. (2015). Serum Concentrations of Kynurenines in Adult Patients with Attention-Deficit Hyperactivity Disorder (ADHD): a Case-Control Study. Behav. Brain Funct. 11, 36. 10.1186/s12993-015-0080-x 26542774 PMC4636001

[B2] Alvarez-Arellano L. Gonzalez-Garcia N. Salazar-Garcia M. Corona J. C. (2020). Antioxidants as a Potential Target against Inflammation and Oxidative Stress in Attention-Deficit/Hyperactivity Disorder. Antioxidants, 9. (Basel). 10.3390/antiox9020176 PMC7070894 32098021

[B3] Anand D. Colpo G. D. Zeni G. Zeni C. P. Teixeira A. L. (2017). Attention-Deficit/Hyperactivity Disorder and Inflammation: What Does Current Knowledge Tell Us? A Systematic Review. Front. Psychiatry 8, 228. 10.3389/fpsyt.2017.00228 29170646 PMC5684106

[B4] Aparicio C. F. Hennigan P. J. Mulligan L. J. Alonso-Alvarez B. (2019). Spontaneously Hypertensive (SHR) Rats Choose More Impulsively Than Wistar-Kyoto (WKY) Rats on a Delay Discounting Task. Behav. Brain Res. 364, 480–493. 10.1016/j.bbr.2017.09.040 28963043

[B5] Bucci D. J. Hopkins M. E. Keene C. S. Sharma M. Orr L. E. (2008). Sex Differences in Learning and Inhibition in Spontaneously Hypertensive Rats. Behav. Brain Res. 187, 27–32. 10.1016/j.bbr.2007.08.022 17904233 PMC2213537

[B6] Calabrese V. Mancuso C. Calvani M. Rizzarelli E. Butterfield D. A. Giuffrida Stella A. M. (2007). Nitric Oxide in the Central Nervous System: Neuroprotection versus Neurotoxicity. Nat. Rev. Neurosci. 8, 766–775. 10.1038/nrn2214 17882254

[B7] Colas D. Gharib A. Bezin L. Morales A. Guidon G. Cespuglio R. (2006). Regional Age-Related Changes in Neuronal Nitric Oxide Synthase (nNOS), Messenger RNA Levels and Activity in SAMP8 Brain. BMC Neurosci. 7, 81. 10.1186/1471-2202-7-81 17184520 PMC1766358

[B8] Corona J. C. (2020). Role of Oxidative Stress and Neuroinflammation in Attention-Deficit/Hyperactivity Disorder. Antioxidants, 9. (Basel). 10.3390/antiox9111039 PMC7690797 33114154

[B9] Domek-Łopacińska K. U. Strosznajder J. B. (2010). Cyclic GMP and Nitric Oxide Synthase in Aging and Alzheimer's Disease. Mol. Neurobiol. 41, 129–137. 20213343 10.1007/s12035-010-8104-x

[B10] Doris P. A. (2017). Genetics of Hypertension: an Assessment of Progress in the Spontaneously Hypertensive Rat. Physiol. Genomics 49, 601–617. 10.1152/physiolgenomics.00065.2017 28916635 PMC5792135

[B11] Dunn G. A. Nigg J. T. Sullivan E. L. (2019). Neuroinflammation as a Risk Factor for Attention Deficit Hyperactivity Disorder. Pharmacol. Biochem. Behav. 182, 22–34. 10.1016/j.pbb.2019.05.005 31103523 PMC6855401

[B12] Ferrari M. F. Fior-Chadi D. R. (2005). Differential Expression of nNOS mRNA and Protein in the Nucleus Tractus Solitarii of Young and Aged Wistar-Kyoto and Spontaneously Hypertensive Rats. J. Hypertens. 23, 1683–1690. 10.1097/01.hjh.0000179163.68634.c3 16093913

[B13] Finch C. E. (2003). Neurons, Glia, and Plasticity in Normal Brain Aging. Neurobiol. Aging 24 (Suppl. 1), S123–S127. 10.1016/s0197-4580(03)00051-4 12829120

[B14] Ganten D. Lindpaintner K. Ganten U. Peters J. Zimmermann F. Bader M. (1991). Transgenic Rats: New Animal Models in Hypertension Research. Invited Lecture. Hypertension 17, 843–855. 10.1161/01.hyp.17.6.843 2045167

[B15] Han S. Rudd J. A. Hu Z. Y. Zhang L. Yew D. T. Fang M. (2010). Analysis of Neuronal Nitric Oxide Synthase Expression and Increasing Astrogliosis in the Brain of Senescence-Accelerated-Prone 8 Mice. Int. J. Neurosci. 120, 602–608. 10.3109/00207454.2010.503911 20707635

[B16] Hojná S. Kadlecová M. Dobešová Z. Valoušková V. Zicha J. Kuneš J. (2007). The Participation of Brain NO Synthase in Blood Pressure Control of Adult Spontaneously Hypertensive Rats. Mol. Cell Biochem. 297, 21–29. 10.1007/s11010-006-9318-0 17009099

[B17] Hussein G. Nakamura M. Zhao Q. Iguchi T. Goto H. Sankawa U. (2005). Antihypertensive and Neuroprotective Effects of Astaxanthin in Experimental Animals. Biol. Pharm. Bull. 28, 47–52. 10.1248/bpb.28.47 15635162

[B18] Kim S. Y. Jin C. Y. Kim C. H. Yoo Y. H. Choi S. H. Kim G. Y. (2019). Isorhamnetin Alleviates Lipopolysaccharide-Induced Inflammatory Responses in BV2 Microglia by Inactivating NF-Κb, Blocking the TLR4 Pathway and Reducing ROS Generation. Int. J. Mol. Med. 43, 682–692. 10.3892/ijmm.2018.3993 30483725 PMC6317673

[B19] Klein M. Onnink M. Van Donkelaar M. Wolfers T. Harich B. Shi Y. (2017). Brain Imaging Genetics in ADHD and beyond - Mapping Pathways from Gene to Disorder at Different Levels of Complexity. Neurosci. Biobehav. Rev. 80, 115–155. 10.1016/j.neubiorev.2017.01.013 28159610 PMC6947924

[B20] Kluknavsky M. Balis P. Puzserova A. Radosinska J. Berenyiova A. Drobna M. (2016). (-)-Epicatechin Prevents Blood Pressure Increase and Reduces Locomotor Hyperactivity in Young Spontaneously Hypertensive Rats. Oxid. Med. Cell Longev. 2016, 6949020. 10.1155/2016/6949020 27885334 PMC5112311

[B21] Law A. Gauthier S. Quirion R. (2003). Alteration of Nitric Oxide Synthase Activity in Young and Aged Apolipoprotein E-Deficient Mice. Neurobiol. Aging 24, 187–190. 10.1016/s0197-4580(02)00068-4 12493565

[B22] Leffa D. T. Panzenhagen A. C. Salvi A. A. Bau C. H. D. Pires G. N. Torres I. L. S. (2019). Systematic Review and Meta-Analysis of the Behavioral Effects of Methylphenidate in the Spontaneously Hypertensive Rat Model of Attention-Deficit/hyperactivity Disorder. Neurosci. Biobehav. Rev. 100, 166–179. 10.1016/j.neubiorev.2019.02.019 30826386

[B23] Leffa D. T. Torres I. L. S. Rohde L. A. (2018). A Review on the Role of Inflammation in Attention-Deficit/Hyperactivity Disorder. Neuroimmunomodulation 25, 328–333. 10.1159/000489635 29874674

[B24] Li Q. Wong J. H. Lu G. Antonio G. E. Yeung D. K. Ng T. B. (20091792). Gene Expression of Synaptosomal-Associated Protein 25 (SNAP-25) in the Prefrontal Cortex of the Spontaneously Hypertensive Rat (SHR). Biochimica Biophysica Acta (BBA) - Mol. Basis Dis. 1792, 766–776. 10.1016/j.bbadis.2009.05.006 19482079

[B25] Lind M. Hayes A. Caprnda M. Petrovic D. Rodrigo L. Kruzliak P. (2017). Inducible Nitric Oxide Synthase: Good or Bad? Biomed. Pharmacother. 93, 370–375. 10.1016/j.biopha.2017.06.036 28651238

[B26] Linnerbauer M. Wheeler M. A. Quintana F. J. (2020). Astrocyte Crosstalk in CNS Inflammation. Neuron 108, 608–622. 10.1016/j.neuron.2020.08.012 32898475 PMC7704785

[B27] Majdak P. Ossyra J. R. Ossyra J. M. Cobert A. J. Hofmann G. C. Tse S. (2016). A New Mouse Model of ADHD for Medication Development. Sci. Rep. 6, 39472. 10.1038/srep39472 27996970 PMC5171883

[B28] Medin T. Medin H. Hefte M. B. Storm-Mathisen J. Bergersen L. H. (2019). Upregulation of the Lactate Transporter Monocarboxylate Transporter 1 at the Blood-Brain Barrier in a Rat Model of Attention-Deficit/hyperactivity Disorder Suggests Hyperactivity Could Be a Form of Self-Treatment. Behav. Brain Res. 360, 279–285. 10.1016/j.bbr.2018.12.023 30550949

[B29] Mittleman B. B. Castellanos F. X. Jacobsen L. K. Rapoport J. L. Swedo S. E. Shearer G. M. (1997). Cerebrospinal Fluid Cytokines in Pediatric Neuropsychiatric Disease. J. Immunol. 159, 2994–2999. 9300724

[B30] Okie S. (2006). ADHD in Adults. N. Engl. J. Med. 354, 2637–2641. 10.1056/nejmp068113 16790695

[B31] Polanczyk G. De Lima M. S. Horta B. L. Biederman J. Rohde L. A. (2007). The Worldwide Prevalence of ADHD: a Systematic Review and Metaregression Analysis. Ajp 164, 942–948. 10.1176/ajp.2007.164.6.942 17541055

[B32] Qadri F. Arens T. Schwarz E.-C. Häuser W. Dendorfer A. Dominiak P. (2003). Brain Nitric Oxide Synthase Activity in Spontaneously Hypertensive Rats during the Development of Hypertension. J. Hypertens. 21, 1687–1694. 10.1097/00004872-200309000-00018 12923401

[B33] Réus G. Z. Fries G. R. Stertz L. Badawy M. Passos I. C. Barichello T. (2015). The Role of Inflammation and Microglial Activation in the Pathophysiology of Psychiatric Disorders. Neuroscience 300, 141–154. 10.1016/j.neuroscience.2015.05.018 25981208

[B34] Rubattu S. Cotugno M. Forte M. Stanzione R. Bianchi F. Madonna M. (2018). Effects of Dual Angiotensin Type 1 Receptor/neprilysin Inhibition vs. Angiotensin Type 1 Receptor Inhibition on Target Organ Injury in the Stroke-Prone Spontaneously Hypertensive Rat. J. Hypertens. 36, 1902–1914. 10.1097/hjh.0000000000001762 29916993

[B35] Russell V. A. (2007). Neurobiology of Animal Models of Attention-Deficit Hyperactivity Disorder. J. Neurosci. Methods 161, 185–198. 10.1016/j.jneumeth.2006.12.005 17275916

[B36] Russell V. A. Oades R. D. Tannock R. Killeen P. R. Auerbach J. G. Johansen E. B. (2006). Response Variability in Attention-Deficit/Hyperactivity Disorder: a Neuronal and Glial Energetics Hypothesis. Behav. Brain Funct. 2, 30. 10.1186/1744-9081-2-30 16925830 PMC1624838

[B37] Song Y. Yuan H. Chen T. Lu M. Lei S. Han X. (2020). An Shen Ding Zhi Ling Alleviates Symptoms of Attention Deficit Hyperactivity Disorder via Anti-inflammatory Effects in Spontaneous Hypertensive Rats. Front. Pharmacol. 11, 617581. 10.3389/fphar.2020.617581 33536923 PMC7847841

[B38] Spiers J. G. Chen H.-J. C. Bourgognon J.-M. Steinert J. R. (2019). Dysregulation of Stress Systems and Nitric Oxide Signaling Underlies Neuronal Dysfunction in Alzheimer's Disease. Free Radic. Biol. Med. 134, 468–483. 10.1016/j.freeradbiomed.2019.01.025 30716433

[B39] Sun G.-C. Wong T.-Y. Chen H.-H. Ho C.-Y. Yeh T.-C. Ho W.-Y. (2019). Angiotensin II Inhibits DDAH1-nNOS Signaling via AT1R and μOR Dimerization to Modulate Blood Pressure Control in the Central Nervous System. Clin. Sci. (Lond) 133, 2401–2413. 10.1042/cs20191005 31755934

[B40] Tayebati S. K. Tomassoni D. Amenta F. (2012). Spontaneously Hypertensive Rat as a Model of Vascular Brain Disorder: Microanatomy, Neurochemistry and Behavior. J. Neurological Sci. 322, 241–249. 10.1016/j.jns.2012.05.047 22726353

[B41] Tomassoni D. Avola R. Di Tullio M. A. Sabbatini M. Vitaioli L. Amenta F. (2004). Increased Expression of Glial Fibrillary Acidic Protein in the Brain of Spontaneously Hypertensive Rats. Clin. Exp. Hypertens. 26, 335–350. 10.1081/ceh-120034138 15195688

[B42] Török J. (2008). Participation of Nitric Oxide in Different Models of Experimental Hypertension. Physiol. Res. 57, 813–825. 10.33549/physiolres.931581 19154086

[B43] Vilskersts R. Kuka J. Liepinsh E. Cirule H. Gulbe A. Kalvinsh I. (2014). Magnesium Nitrate Attenuates Blood Pressure Rise in SHR Rats. Magnes. Res. 27, 16–24. 10.1684/mrh.2014.0358 24827813

[B44] Volke V. Soosaar A. Ko˜ks S. Bourin M. Männistö P. T. Vasar E. (1997). 7-Nitroindazole, a Nitric Oxide Synthase Inhibitor, Has Anxiolytic-like Properties in Exploratory Models of Anxiety. Psychopharmacology 131, 399–405. 10.1007/s002130050309 9226743

[B45] Wu Y. Zhang A. Yew D. (2005). Age Related Changes of Various Markers of Astrocytes in Senescence-Accelerated Mice hippocampus. Neurochem. Int. 46, 565–574. 10.1016/j.neuint.2005.01.002 15843051

[B46] Yokokura M. Takebasashi K. Takao A. Nakaizumi K. Yoshikawa E. Futatsubashi M. (2021). *In Vivo* imaging of Dopamine D1 Receptor and Activated Microglia in Attention-Deficit/hyperactivity Disorder: a Positron Emission Tomography Study. Mol. Psychiatry 26, 4958–4967. 10.1038/s41380-020-0784-7 32439845

